# Evaluating user enjoyment, engagement, and intention to play in edugames4all MicrobeQuest!

**DOI:** 10.3389/fpubh.2026.1789009

**Published:** 2026-07-27

**Authors:** Andreea Molnar, Patty Kostkova

**Affiliations:** 1 Department of Computing Technology, Swinburne University of Technology, Hawthorn, VIC, Australia; 2 UCL Centre for Digital Public Health in Emergencies, Department for Risk & Disaster Reduction, and Collaborating Centre for Digital Public Health and Pandemic Preparedness, University College London, London, United Kingdom

**Keywords:** AMR, antibiotic resistance, engagement, enjoyment, games for health, hand washing, hygiene, intention to play

## Abstract

**Background:**

The widespread use of mobile phones among children and the growing popularity of games in this age group have led to the development of mobile learning applications, including educational health games. However, many of these games either fail to present healthcare topics in an engaging way for children or are still in early development stages. Although there is increasing research interest in serious educational health games for children, their appeal, such as enjoyment, engagement, and intention to play, is often not properly evaluated. However enjoyment is what motivates the use of games for learning especially for children, and educational games must be engaging and rewarding, as this fosters greater persistence, achievement, and completion outcomes among students.

**Methods:**

This research presents an evaluation of the appeal of a mobile educational game, Edugames4all MicrobeQuest!, designed to promote hygiene and responsible antibiotic use among primary school children. The evaluation focuses on three dimensions of appeal: enjoyment, engagement, and intention to play, and relationship between them. The study included 19 participants.

**Results:**

The findings indicate that the game was generally appealing to children across all three evaluated dimensions. It also shows a strong and positive correlation between enjoyment, engagement and intention to play.

**Conclusions:**

Evaluating the appeal of educational health games is important, as it cannot be assumed that children will enjoy them simply because they are games. The study shows that the evaluated game successfully achieved a good level of appeal among its target audience. The three constructs are positively correlated.

## 1 Introduction

Without effective antibiotics, even routine surgeries could become dangerously risky to perform. The Global Antibiotic Resistance Surveillance Report 2025 (1) shows a 40% increase in antibiotic resistance for the monitored antibiotics between 2018 and 2023. Using antibiotics optimally and reducing the need to use them through adequate hand hygiene can help slow antibiotic resistance (2). In addition, for children, interventions that aim to improve hand hygiene have been shown to significantly reduce some illness-related school absences (3). Therefore, empowering children with knowledge of hygiene and responsible use of antibiotics could help alleviate some absences related to illness, reducing antibiotic resistance but also promoting more responsible use of antibiotics as they become adults (4).

When designing educational activities for children, it is important that they are engaging. Games have the potential to create engaging and enjoyable experiences (5). However, educational games are sometimes criticized for being “chocolate-covered broccoli" (6) by attempting to make inherently unappealing educational content more engaging by adding superficial elements of fun, such as points, rewards. Adding entertainment as a layer over the educational content does not always result in meaningful engagement. Players, as a result, perceive the disconnect between the “chocolate" (game-like features) and the “broccoli" (educational content).

Therefore, in addition to learning, making sure that the player finds the experience of playing the game enjoyable needs to be considered when using the game, as the fact that the games are enjoyable is the reason why a game would be chosen over a less expensive non-gaming learning environment (7). In addition, according to Csikszentmihalyi et al. (8), “learning has to be engaging and rewarding for students to learn" (p. 9). This has been linked to many benefits in education, such as students obtaining higher grades, being less likely to give up, and having higher completion rates (9, 10).

Currently, there is an increase in the ownership of mobile devices among children and young adults (11, 12), with most children in the developed world having access to a smartphone (13). Moreover, mobile devices are more common among children than laptops or personal computers (14). Taking this into account, our study focuses on mobile educational games designed for children, specifically those aimed at promoting knowledge about hygiene practices and antibiotic resistance. There are several educational games aimed at children and focusing on hygiene and/or responsible antibiotic use (15). Among these, edugames4all MicrobeQuest! (16) is also accessible on mobile devices. The game is designed according to learning theories (15). In addition, the game has the advantage that an evaluation of usability (17) and educational potential of the game has already been performed (16), allowing us to focus on the appeal of the game. In the context of this research, the game appeal is measured across three dimensions: enjoyment, engagement, and intention to play. Intention to play shows that the player wants to return to the game, overcoming the “novelty effect." Engagement allows the player to practice for an extended period of time, while enjoyment is the initial “affective hook" creating a positive emotional state that increases intrinsic motivation (18). As a result, the research questions this study aims to answer are: “*What is the appeal of the mobile game edugames4all Microbequest! for primary school children?"* and *How are enjoyment, engagement, and intention to play related?*

## 2 Materials and methods

The purpose of the study was to evaluate the Edugames4all MicrobeQuest!'s game appeal for the children playing it, with the aim of determining whether the game goes beyond just delivering the educational message. We do not aim to provide a generalization, as this is not possible, but to determine whether the game is appealing to the children. The game's appeal was defined in the context of this research as measuring the children's enjoyment, engagement, and intention to play similar games. We also measure the correlation between these three constructs. Due to the small sample size (*N* = 19), descriptive statistics (19) were used to display the results of the appeal of the game and Spearman's rank correlation (20) to determine the relationship between them.

### 2.1 Instruments

#### 2.1.1 Questionnaire

The questionnaires were adapted from Wrzesien and Raya (21) that measured the same constructs for children of similar age. Table 1 presents the original questions and the adapted one divided by the constructs. Each dimension is assessed through three questions. The participants had to select how much they agreed with a statement on a 5-point Likert scale, labeled: *Not at all (1), A little (2), Quite a lot (3), A lot (4)* and *Very (5)*. Cronbach's alpha (22) was computed for each construct to determine internal consistency (see the last column of Table 1). For the *Enjoyment* and *Intention to play* constructs, a value over 0.70 was obtained, which is considered the threshold for acceptable internal consistency (23). For *Engagement* the Cronbach's alpha value is 0.69, just below the threshold. Although a Cronbach's alpha value of 0.70 is commonly recommended, lower values can be acceptable for scales with a small number of items (as this scale is), as Cronbach's alpha value is sensitive to test length (i.e., a lower number of items tends to get lower results) (23, 24).

**Table 1:** Questionnaire.

Construct	Original question	Adapted question	Cronbach's alpha
Enjoyment	This type of class is fun	This type of game is fun	0.85
	I had fun during this type of class	I had fun playing this game	
	It is nice to participate in this type of class	It is nice to play this game	
Engagement	I forgot about time passing while participating in the class	I forgot about time passing while playing the game	0.69
	I become unaware of my surroundings while participating in this type of class	I become unaware of my surroundings while playing the game	
	I temporally forget worries about everyday life while I participated in this type of class	I temporarely forget about worries about everyday life while playing the game	
Intention to play	Based on my experience, I would like to participate more in this type of class	Based on my experience, I would like to learn by playing a similar game	0.75
	I want to participate more in this type of class as soon as it possible	I want to learn by playing this kind of game as soon as possible	
	If it would be possible I would like to participate in this type of class shortly	If it would be possible I would like to replay this game shortly	

#### 2.1.2 The game: Edugames4all MicrobeQuest!

**Figure 1:**
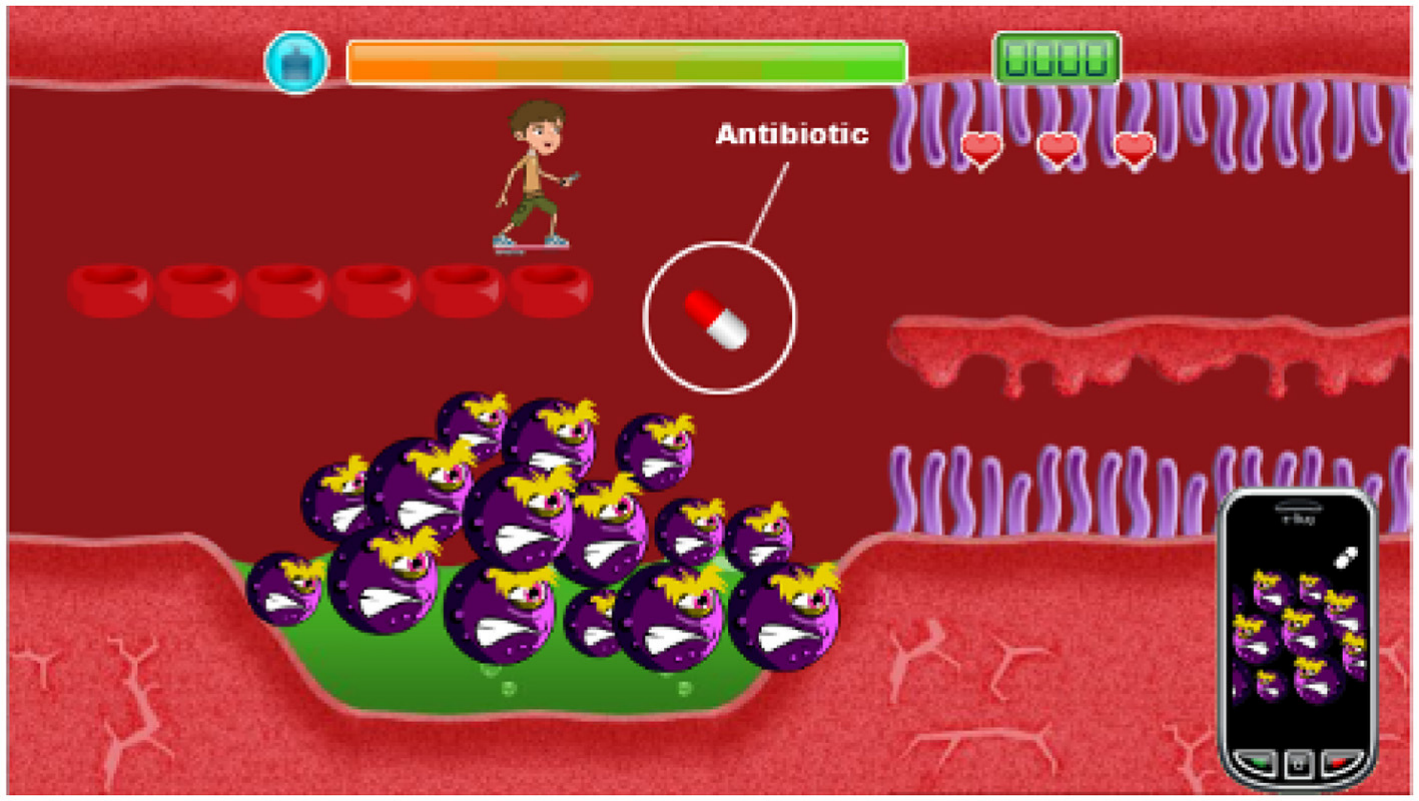
Player avatar inside the human body throwing antibiotics (15).

Edugames4all MicrobeQuest! (16) is an educational game aimed at teaching primary school children (9–12 years old) about hygiene and responsible use of antibiotics. The game has five levels: (1) Introduction to Microbes, (2) Harmful Microbes, (3) Useful Microbes, (4) Food hygiene, and (5) Antibiotics. The learning objectives covered in these levels were selected to meet what it is taught in the primary school curriculum in Europe (25). These are integrated through a combination of text and game mechanics (26). An example of integration with game mechanics consists of the player (shrunk to the level of a microorganism and exploring the human body) throwing antibiotics at the bacteria found in the body (see Figure 1). If the player does not deliver the full course of antibiotics, the bacteria becomes stronger. This aims to teach children about following the prescribed course of treatment. For children who are not familiar with this game genre, there is also a tutorial mission which introduces the game mechanics. From a pedagogical perspective, the game is designed around a constructivist approach (27), drawing on the ideas of Koster (28) and Shaffer and Gee (29). The mechanics of the game are used as a support for learning, and all learning objectives are constructively aligned.

From a design lens, Mechanics-Dynamics-Aesthetics (MDA) framework (30) groups the elements of a game around three core components: Mechanics (rules on how the game operates), Dynamics (how the Mechanics interact based on user input), and Aesthetics (player emotional response). In Edugames4all Microbequest! some example of mechanics are:

- Avatar interaction: the player avatar shrinks to the size of a microorganism. This happens at every level except for *Food hygiene*, where the avatar is in the kitchen. This enables direct interaction with the environment. See, for example, Figure 1 for the player's avatar inside the human body. This mechanic encourages immersion and perspective-taking. By being immersed in the environment, the players can understand how common microorganisms are and learn by seeing and interacting with these microorganisms.
- Exploration and navigation of different environments (e.g., human hand, human body): different levels of the game focus on a certain environment. This serves as a discovery-based learning (31), with players being able to observe environmental differences, identify microorganisms (the design of the microorganism present in the game is done so that it is both pleasant for children and also designed to look close to how the microorganisms look), and learn about their interaction with specific external influences (e.g., administering antibiotics, using soap to wash hands). The navigation system also supports player's agency and curiosity, allowing learners to actively engage with educational content rather than passively receiving information.
- Levels and task based progression: the players have five levels to complete, each focused on an environment. To complete each level, they have to solve challenges such as using antibiotics to fight infections, soap to wash hands. At the end and beginning of each level, there is a quiz structured in a way similar to a “How to be a millionaire" show (32). During this part, the player is competing with another player in answering educational questions. This mechanic creates a rule and reward structure while explicitly reinforcing in the quiz the knowledge accumulated through the game mechanics. The goal-oriented approach provides engagement and motivation.
- Feedback: the game incorporates both explicit and implicit feedback mechanisms. Explicit feedback is provided through text-based explanations (26), and instructional prompts after the actions of the player or the responses to the questions. Implicit feedback emerges through gameplay consequences and environmental interactions, such as successful progression after correct decisions or restricted advancement following incorrect choices. These feedback loops support learning reinforcement, self-assessment, and iterative improvement while maintaining player engagement.

Players' engage with mechanics through play (the dynamics component of the framework). They discover and learn through exploration (as the player explores the human body, they encounter microorganisms and learn about them). Their progression through the game is goal oriented, they having to complete specific tasks to complete the mission (e.g., finishing the course of antibiotics) and answer questions about educational content covered at the end of each mission. The game allows for iterative learning to happen by players' learning through repeated actions and formative feedback.

The aesthetic dimension of MicrobeQuest! centers on the experiential and emotional outcomes of game play. The game is designed to evoke curiosity and discovery (through discovering microorganism and where they can be found), enjoyment and engagement (through challenges and progression), confidence in learning (through feedback and completing tasks). Visual design elements–such as colorful representations of microbes and intuitive interfaces–further support accessibility and appeal, particularly for younger learners.

The previous versions of the game were evaluated both for educational potential (16, 33) and usability (32). The game has both a mobile (16) and a desktop version (33). We used the mobile version of this game for evaluation purposes.

### 2.2 Participants

A convenience sampling method (34) was used to recruit participants. A total of 19 participants were recruited on a voluntary basis from a school and a computer club program in the UK. Their average age was 9.2 years. Most of them were playing games on a daily basis. A total of eight participants were male and 11 female. The sample size is in line with sample sizes used in similar human computer interaction studies (35). According to Caine (35), the most commonly reported sample size in the human-computer interaction community is 12, with in-person studies having relatively small sample sizes due to practical constraints. However, there are limitations when working with small sample sizes, which we acknowledge and discuss in Section 4.2.

### 2.3 Set-up and methodology

Due to the age of the participants we were recruiting, written consent was obtained from the parents and the school/after-school program. Before giving consent, written information about the study was provided to the organizations that facilitate the study and to parents. Before the study, the children were informed what they were expected to do in the study and offered the opportunity to withdraw. They were also told that they could withdraw at any stage during the study. Verbal consent was obtained from the participants (none of the participants chose to withdraw).

The study follows the following steps:

- The participants were informed about the study and consent was obtained.
- Afterwards they had to fill a short questionnaire collecting information such as demographic data (age, gender), how often they play games
- The participants were provided with a mobile phone with the game already installed and were asked to play the game for 30 min. All the participants played the game from the beginning; however, they advanced through the game at their own pace (i.e., not all the participants would have finished the game by the end of the session).
- At the end of the game playing session, they were asked to fill in a second questionnaire evaluating the game

All participants followed the above steps, and a researcher was present during the study.

The questionnaires were previously used in Wrzesien and Raya (21), and the questions were either used verbatim or adapted to our context [i.e., the questionnaire in Wrzesien and Raya (21) measured the intention to participate in a similar type of class, which, in our case, the wording has changed to intention to play the game]. This modification is unlikely to affect the underlying construct being evaluated, as minor contextual wording changes do not necessarily alter construct validity when items retain their conceptual meaning. This is supported by measurement invariance theory, which suggests that instruments can remain valid across contexts provided that the items are interpreted equivalently (36). Data was collected using paper questionnaires. This was later on introduced into a CSV sheet, stored on the university server, and analyzed.

## 3 Results

The game appeal was defined across three dimensions: enjoyment, engagement, and intention to play similar games. The results across each of these three aspects are presented below in the first subsection, which answers the research question 1: “*What is the appeal of the mobile game edugames4all Microbequest! for primary school children?"* The last subsection provides an answer to research question 2: *How are engagement, intention to play, and enjoyment related?*

### 3.1 Game appeal

#### 3.1.1 Enjoyment

**Figure 2:**
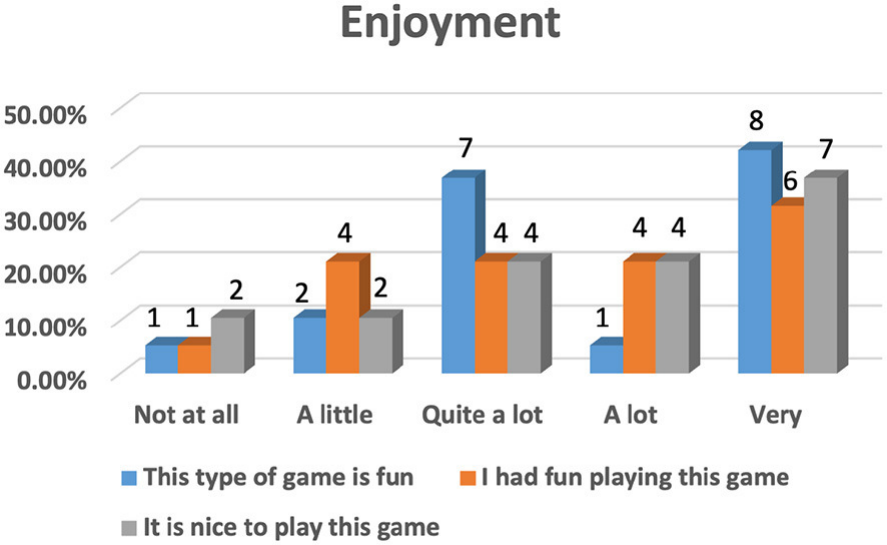
Player's enjoyment.

Figure 2 provides a detailed overview of the percentage of participants selecting a certain option for each question and, on top of the bars, the number of participants choosing the given option. A total of 95% of the participants selected either *Quite a lot, A lot, Very* or *A little* when asked if the game is fun and that they had fun while playing the game, while 89% felt that it was to a certain extent nice to play the game. Overall, very few of the participants did not enjoy at all playing the game. Table 2 presents the mean and standard deviation for each question. The mean indicates a consistent high level of enjoyment (between *Quite a lot* and *A lot*) across all three questions. The standard deviation, just above one, suggests that the players' experience with the game differs.

**Table 2:** Mean and standard deviation for the game appeal concepts.

Concept	Mean	Std. deviation
Enjoyment	3.57	1.19
Engagement	2.82	1.18
Intention to play	3.25	1.15

#### 3.1.2 Engagement

**Figure 3:**
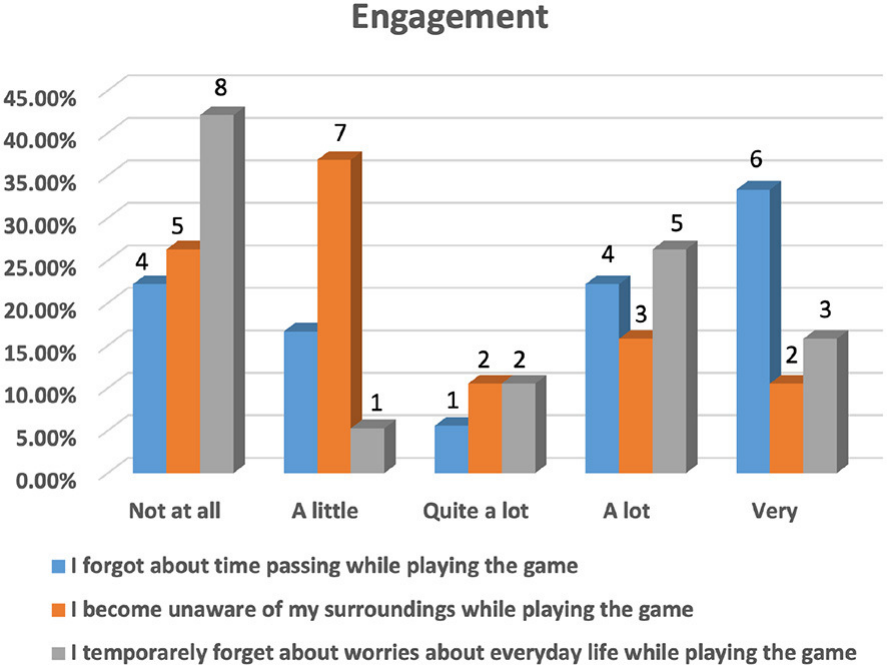
Player's engagement.

Figure 3 provides a detailed overview of the percentage of participants selecting a certain option for each question and, on top of the bars, the number of participants choosing the given option. Most of the participants selected *Quite a lot, A lot, Very* or *A little* in all three questions. Most of them forgot about the time passing (78%) and their surroundings (74%) while playing the game. A total of 58% reported forgetting to a certain extent about their worries while playing the game. Table 3 presents the mean and standard deviation for each question. The mean for the first question *I forgot about time passing while playing the game* is 3.28 indicating that the game holds the participants' attention. For the other two questions, the mean is lower, suggesting that the players were aware of their environment and had a moderate level of immersion. The high standard deviation (between 1.35 and 2.68)—especially for the last question, *I temporarily forget about worries about everyday life while playing the game*—shows that players have different experiences with the game.

**Table 3:** Mean and standard deviation for engagement questions.

Question	Mean	Std. deviation
I forgot about time passing while playing the game	3.28	1.64
I become unaware of my surroundings while playing the game	2.47	1.35
I temporarily forget about worries about everyday life while playing the game	2.68	1.64

#### 3.1.3 Intention to play

**Figure 4:**
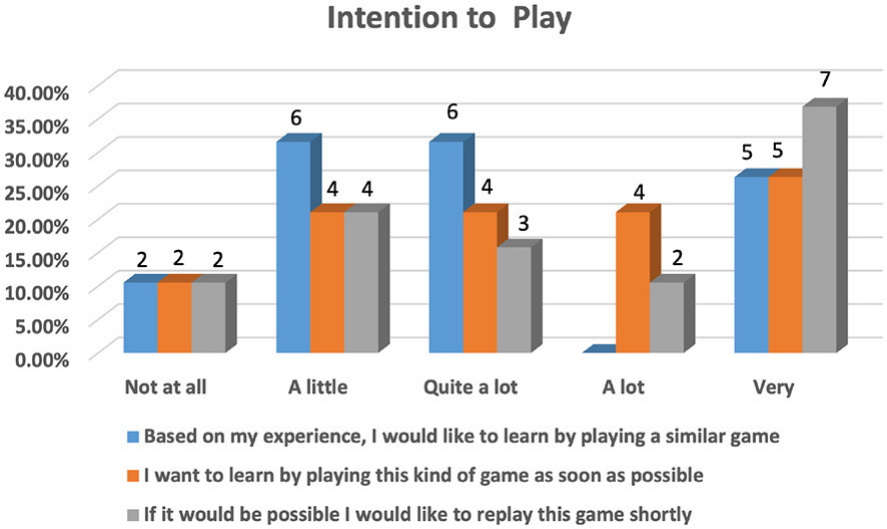
Intention to play.

Figure 4 provides a detailed overview of the percentage of participants selecting a certain option for each question and, on top of the bars, the number of parti4cipants choosing the given option. Very few participants (11%) selected *Not at All* in any of the three options. Therefore, most of the participants felt to a certain degree that they would like to play a similar game, learn by playing this kind of game, and replay this game. The percentage of people who wanted to replay is slightly lower than for the other two questions. Table 4 presents the mean and standard deviation for each question. The mean on all the questions exceeds the *Quite a lot (3)* threshold showing a strong intention to play across all three dimensions. The standard deviation slightly above one for all questions suggests that while participants were eager to play the game, there were also some who were not that interested.

**Table 4:** Mean and standard deviation for intention to play questions.

Question	Mean	Std. deviation
Based on my experience, I would like to learn by playing a similar game	3.00	1.37
I want to learn by playing this kind of game as soon as possible	3.32	1.37
If it would be possible I would like to replay this game shortly	3.42	1.46

#### 3.1.4 Summary

Table 5 presents the mean and standard deviation for each concept. Among the three concepts, *Enjoyment* received the highest mean score (*M* = 3.57), placing it between *Quite a lot* (3) and *A lot* (4), suggesting that participants found the game enjoyable. *Intention to Play* has a mean of 3.25, also sitting near the *Quite a lot anchor*. *Engagement* scored the lowest of the three (*M* = 2.82), falling just below the *Quite a lot* point, showing that participants felt a limited degree of engagement with the game. The standard deviation ranges from 1.15 to 1.19, reflecting a similar level of variability in responses and suggesting that players had different experiences with the game.

**Table 5:** Mean and standard deviation for enjoyment questions.

Question	Mean	Std. deviation
This type of game is fun	3.68	1.29
I had fun playing this game	3.53	1.30
It is nice to play this game	3.63	1.38

### 3.2 Connection between engagement, intention to play and enjoyment

Spearman's (37) correlation analysis shows significant positive relationships among enjoyment, engagement, and intention to play, suggesting that all three components are strongly connected. Intention to play was strongly correlated with enjoyment (*r* = 0.829, *p* < 0.001) and engagement (*r* = 0.723, *p* < 0.001). Engagement was also positively associated with enjoyment (*r* = 0.675, *p* < 0.001).

## 4 Discussions

### 4.1 Results in broad context

Overall, for most of the study participants, the enjoyment was high. Most felt that they would like to learn using a similar game, and for most of the participants, the game was engaging. The informal feedback received by the researcher who conducted the study was that the players enjoyed the game and were looking forward to participating in other studies.

However, there were a few participants who reported that the game appeal was low. This could be explained by the fact that not all children would have been familiar with or would enjoy playing this type of game genre. Previous research has shown that compatibility (which refers to personality traits and the situation in which the mobile is used) could affect engagement with mobile apps (38). Moreover, traditional education is more effective for children/young adults who do not usually play video games, and the gaming profile could affect interest and motivation to learn with a certain type of game (39).

Few participants reported that they would not play a similar type of game again. This could be explained by the fact that the game had fewer levels than a typical commercial game, making replaying it less attractive to children, as game levels contribute to a sense of progression and achievement (40).

The correlation analysis shows strong and statistically significant positive relationships between enjoyment, engagement, and intention to play. In particular, intention to play showed a strong association with enjoyment (*r* = 0.829, *p* < 0.001), indicating that participants who reported greater enjoyment were substantially more likely to express a stronger intention to continue or replay the game. This finding aligns with previous research suggesting that enjoyment is one of the motivational factors driving continued gameplay behavior and user retention in digital games and interactive systems (41, 42). Furthermore, the relationship between engagement and intention to play highlights the importance of designing interactive experiences that promote immersion and sustained cognitive involvement, both of which are associated with deeper learning and knowledge retention in educational games (45).

The high enjoyment scores suggest that the game is effective in attracting players, which is useful for the initial adoption and engagement with the topic. Previous research shows a positive correlation between engaging learning experiences and learning (9, 10, 43). In addition, for some players, the game leads to a state of flow. This suggests a low mental burden that leads to people more likely to repeat the experience (44), as our results also show. Engaging learning are correlated with increased learning (45), and engaging learning experiences such as this game could help children engage with health topics. Exposure to this information is a first step in behavior change, and better hygiene, such as hand washing, reduces illness and school absenteism related to illness (3), as well as prescription of antibiotics (46). In addition, children are the future prescribes and users of antibiotics, and knowledge of appropriate antibiotic use could reduce inappropriate use and, consequently, antibiotic resistance. However, this study only shows that the game has been engaging and did not study how this influences learning and behavior change. Future research should consider whether it can also apply to this particular game. Although there is research that supports the positive correlation between engaging experiences and learning (47) shows that a focus on maximizing engagement could reduce the efficacy of clinical interventions. Healthcare interventions using games or gamification should consider how to carefully balance these aspects.

### 4.2 Limitations

Although the sample size is similar to other human computer interaction studies (35), the study used a relatively small sample, consisting exclusively of students based in metropolitan areas within the United Kingdom. This limited sample size and geographic concentration may restrict the generalisability of the findings, as it does not account for potential variations present in rural or non-UK populations. Consequently, the results should be interpreted in this specific context. In addition, our study uses a single game that was played for 30 min. These could have influenced how the players perceived the game appeal, and the strong positive correlation between the game appeal components.

To enhance the robustness and external validity of future research, subsequent studies should aim to recruit a larger and more diverse sample by expanding participation to include students from different regions, educational backgrounds, and demographic groups. This would strengthen the applicability of the findings in wider contexts. Additionally, studies that include different educational games should be used to determine replicability of the relationship between enjoyment, engagement, and intention to play.

## 5 Conclusions

Educational games can enhance children's learning by creating enjoyable and engaging experiences, which can lead to children engagement with the topic, increased learning and persistence. However, simply adding game elements to educational content may not result in enjoyable learning experiences. This research aimed to assess the game appeal of a mobile game for education. We used as a case study edugames4all MicrobeQuest!, a mobile game aimed at teaching children microbiology. The game uses a combination of text and game mechanics to teach and reinforce the learning objectives. The learning objectives covered in the game are based on the primary school biology curriculum. Building on previous statistical evaluation of knowledge gain (16) this paper presented the results of a study assessing the game's appeal for primary school children. The game's appeal was measured across three dimensions: enjoyment, engagement and intention to play. Most children enjoyed the game a lot and felt that they would like to learn using a similar game. They also reported forgetting about their worries, everyday life, or their surroundings during the play. Overall, most participants felt that the game was appealing. We also found a strong positive correlation among these three concepts.

## Data Availability

The raw data supporting the conclusions of this article will be made available by the authors, without undue reservation.
